# RNA polymerase II pausing in development: orchestrating transcription

**DOI:** 10.1098/rsob.210220

**Published:** 2022-01-05

**Authors:** Abderhman Abuhashem, Vidur Garg, Anna-Katerina Hadjantonakis

**Affiliations:** ^1^ Developmental Biology Program, Sloan Kettering Institute, Memorial Sloan Kettering Cancer Center, New York, NY 10065, USA; ^2^ Weill Cornell/Rockefeller/Sloan Kettering Tri-Institutional MD-PhD Program, New York, NY 10021, USA; ^3^ Biochemistry, Cell and Molecular Biology Graduate Program, Weill Cornell Medical College, New York, NY 10021, USA

**Keywords:** transcription, Pol II pausing, development, embryonic stem cells

## Abstract

The coordinated regulation of transcriptional networks underpins cellular identity and developmental progression. RNA polymerase II promoter-proximal pausing (Pol II pausing) is a prevalent mechanism by which cells can control and synchronize transcription. Pol II pausing regulates the productive elongation step of transcription at key genes downstream of a variety of signalling pathways, such as FGF and Nodal. Recent advances in our understanding of the Pol II pausing machinery and its role in transcription call for an assessment of these findings within the context of development. In this review, we discuss our current understanding of the molecular basis of Pol II pausing and its function during organismal development. By critically assessing the tools used to study this process we conclude that combining recently developed genomics approaches with refined perturbation systems has the potential to expand our understanding of Pol II pausing mechanistically and functionally in the context of development and beyond.

## Graphical abstract

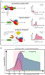

## 1. Introduction

Multicellular organisms rely on differential gene expression to diversify cell types with distinct functions [1,2]. A range of mechanisms have therefore evolved for spatio-temporal regulation of transcription, such as signalling via extracellular or intracellular ligands [1–3]. These pathways eventually regulate activity of nuclear-localized transcription factors that bind to specific DNA *cis*-regulatory elements and initiate or prohibit recruitment of transcriptional machinery to target gene promoters [1].

Over the past few decades, our cumulative understanding of the biochemical basis of transcription has revealed that it does not function as a simple ‘on-off’ switch, but instead involves a sequence of steps that each contribute to the overall transcription rate [1,4–6]. Broadly, gene transcription requires the assembly of a pre-initiation complex (PIC), initiation of transcription, induction of productive elongation, followed by termination. Each of these steps can be regulated by canonical signalling pathways, while classic examples of such regulation include alternative splicing and premature termination and degradation [7–10]. Over the past decade, RNA polymerase II (Pol II) has been shown to accumulate downstream of the transcription start site at certain genes in higher metazoans, possibly to prepare or ‘poise’ promoters for imminent activation [11–13]. This phenomenon, referred to as promoter-proximal Pol II pausing (hereafter referred to simply as ‘Pol II pausing’), is widespread in mammalian transcription, with some studies estimating that up to 40% of protein-coding genes experience pausing [14,15].

The molecular mechanisms responsible for establishing and preserving Pol II pausing have been extensively studied over the last two decades. Genetic ablation of various components of the Pol II pausing machinery has determined that it is required for cell proliferation *in vitro*, for the mid-blastula transition (MBT) in fruit flies, hematopoietic stem cell specification in zebrafish and for early embryo development in mouse [15–18]. These results highlight the evolutionary conservation of Pol II pausing across metazoans [15,19]. Intriguingly, these embryonic phenotypes occur at critical periods of cell fate specification during development. Despite the discovery that Pol II pausing has an essential role in organismal development, mechanistic insight into its specific function remains unclear due to complex and confounding phenotypes in loss-of-function models, and technical difficulties in assessing the primary defects to study the direct impact of disrupting Pol II pausing [12]. Additionally, the majority of studies have been carried out using *in vitro* cell culture models or invertebrate (*Drosophila melanogaster)* embryos, while relatively little is known about the specific roles of Pol II pausing in mammals.

In a mammalian system, an extensive analysis of the Pol II landscape in early pre-implantation mouse development was recently reported, which revealed dynamic pausing at distinct stages between the zygote and early blastocyst stages [20]. Pre-implantation development spans the period from formation of the totipotent fertilized zygote to the blastocyst, just prior to embryo implantation into the maternal uterine wall [21–23]. The blastocyst consists of three cell types that are generated via two binary cell fate decisions [24]. The first of these segregating the trophectoderm (TE) lineage (an extraembryonic lineage and precursor of the fetal placenta) from the inner cell mass (ICM). The second lineage decision specifies the ICM into the pluripotent embryonic epiblast (EPI) and the extraembryonic or primitive, endoderm (PrE; precursor of the yolk sac endoderm). The ability to derive representative stem cell lines from the three blastocyst lineages, particularly the EPI, the source of pluripotent mouse embryonic stem (mES) cells, facilitates study of embryos at this stage. mES cells present an invaluable model to study Pol II pausing in a mammalian system, and have led to several proposed functions of Pol II pausing in mammalian development, such as regulating inputs from signalling pathways, activities of key transcription factors and ultimately, differentiation potential [18,25–27].

In this review, we focus on Pol II pausing from a functional perspective during embryo development. We start by briefly discussing our molecular understanding of Pol II pausing and highlight recent discoveries. We then discuss the established roles for Pol II pausing in different developmental models, with a focus on early mammalian development, and the current limitations and open questions in the field. Finally, we discuss how these limitations could be overcome to gain a deeper understanding of the functional relevance of Pol II pausing during development.

## 2. Pol II pausing: molecular mechanisms and function

### 2.1. Pol II pausing and pause-release

**Figure 1.**
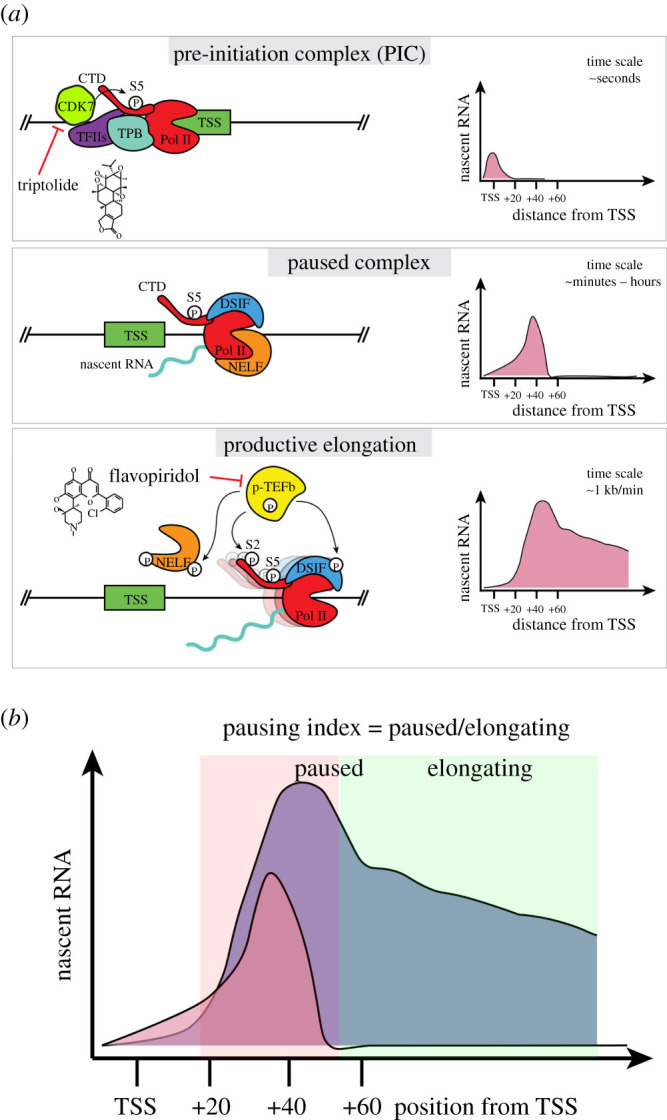
Stepwise activation of mammalian transcription. (*a*) Illustration of three steps leading to active gene transcription along with key complexes involved and example profile of nascent transcription levels along the gene body. (*b*) Illustration of pausing index, a commonly used metric to determine degree of Pol II pausing at a particular gene.

Promoter-proximal RNA polymerase II pausing is defined as a transcription halt following initiation but prior to elongation [12,13]. Following the assembly of the PIC at the transcriptional start site (TSS), transcription is initiated and the polymerase transcribes approximately 20–60 nucleotides. At a subset of promoters, RNA Pol II pauses at this site before proceeding into productive elongation (figure 1). Currently, it remains poorly understood why pausing occurs at certain promoters, particularly in mammals [28]. Pol II pausing is mediated by two major complexes—the negative elongation factor (NELF) and DRB sensitivity inducing factor (DSIF) [29]. Both complexes are highly conserved across metazoans, and have been shown to interact directly with RNA Pol II [30–33]. Recently, these interactions have been visualized at atomic resolution in cryo-EM structures of the complete paused Pol II–DSIF–NELF complex, providing key insights into the mechanism of Pol II pausing [32,33]. Specifically, the bound multi-protein complex tilts the DNA–RNA hybrid, thus impairing the addition of new nucleotides, and preventing the interaction of other pro-elongation factors on Pol II, which is required for productive elongation [33].

For Pol II to proceed to elongation and transcription of the remaining gene body, the paused complex must be released. Paused Pol II is released by an array of general elongation effectors such as BRD4, TRIM33 and the Mediators which ultimately recruit the positive transcription elongation factor, or P-TEFb [12,34–36]. The P-TEFb catalytic subunit CDK9 can phosphorylate paused Pol II, NELFA, NELFE and DSIF, causing their dissociation and elongation to ensue [29] (figure 1). The P-TEFb complex is also responsible for phosphorylating the serine 2 (S2) on the C-terminal domain (CTD) of Pol II and recruiting additional elongation factors, such as the super elongation complex (SEC) [37]. In the absence of pause-release, paused Pol II can result in early termination of a transcript in a context-dependent manner [38]. It is crucial to mention that activating a paused gene does not decrease pausing, but rather controls the rate of release by inducing signals [39]. Given the complex set of interactions between several protein complexes, it is important to note that genetic ablation of single factors or inhibiting their functions does not always provide a straightforward understanding of their impact on the establishment and/or release of paused Pol II. For example, CDK9 is involved in releasing the paused Pol II and promoting elongation, and it is challenging to uncouple the impact on these two processes when inhibiting CDK9 [37].

### 2.2. How prevalent is pausing?

Pol II pausing was initially discovered on heat shock responsive genes in Schneider line 2 (S2) cells derived from *D. melanogaster* embryos [40]. Paused Pol II was similarly shown to accumulate at the human HSP70 family of genes as a means to regulate their expression [41,42]. Thus, heat shock genes in human and *Drosophila* were used to characterize the determinants of, and factors involved in, Pol II pausing [43,44]. Recent advances in genome-wide chromatin capture and nascent RNA sequencing techniques have revealed that Pol II pausing occurs more broadly across the genome. Chromatin immunoprecipitation followed by next generation sequencing (ChIP-seq) and global, or precision nuclear run-on sequencing (GRO/PRO-seq) have facilitated pinpointing the location of transcriptionally engaged Pol II genome-wide at single base resolution [29,45]. These approaches have revealed that Pol II pausing, determined as an accumulation of Pol II signal briefly following the TSS, is widespread on metazoan genes [46,47], ranging from 30% to 70% of the expressed genome among different studies [14,15,48]. This variance in the reported prevalence of pausing can, at least in part, be attributed to the fact that there is no consensus on how to quantitatively define Pol II pausing. While most studies deduce a ‘pausing index’—the ratio of paused to elongating Pol II—the exact genomic range for a polymerase to be considered paused or elongating differs between studies (figure 1). Still, together these data demonstrate that a significant portion of assembled Pol II can be captured in a paused state.

To interrogate the dynamics of Pol II pausing, acute perturbation of transcription initiation or elongation have been carried out using chemical inhibitors such as triptolide and flavopiridol, respectively [49–51]. These small molecules inhibit transcription kinases, CDK7 (triptolide) and CDK9 (flavopiridol) [37]. In *Drosophila* Kc167 cells, inhibition of transcriptional elongation using flavopiridol results in all active genes accumulating paused polymerases, while inhibiting initiation revealed a clear difference in the stability of paused complexes at individual gene loci (ranging from minutes to hours) [27,51]. These experiments suggest that pausing, representing at least a transient step, is present at most active genes. Thus, transcription at most loci would start with PIC assembly, followed by a paused complex 20–60 nucleotides downstream, then proceed to elongation [52]. Overall, these studies suggest that Pol II pausing is a universal step during transcription, yet the rate of pause–release determines if it is a bottleneck step at a given gene. Therefore, Pol II pausing may serve as a rate-limiting step of transcription that can serve as a significant point of transcriptional regulation [52].

### 2.3. Effects of pausing on transcription

While Pol II pausing has been implicated as a key rate-limiting step in transcription, only recently has clear molecular and causal evidence for such a role been demonstrated, highlighted by two key findings. First, by using a combination of improved ChIP- and nascent RNA-seq approaches to locate RNA Pol II on the genome and determine nascent transcriptional output, Gressel *et al*. and Shao *et al*. showed that Pol II pausing inhibits new PIC assembly [49,51]. Cryo-EM structures of the paused Pol II complex strongly suggest that this inhibition is mediated directly via steric hindrance [33]. Second, subsequent work has shown that the paused complex is significantly more stable than other transcriptional steps [50]. Specifically, the PIC is stable for a time scale of seconds and productive elongation occurs at a rate of approximately 1 kb min^−1^, while paused Pol II can be stable for 1–10 min on average, and as long as one hour at some promoters [51].

Of note, the stability, dynamics and turnover of paused Pol II at protein-coding genes has been studied by several groups, albeit with some discrepancy regarding the exact time estimates [38,50,51,53–55]. These studies used genome-wide chromatin capture techniques, genome-wide footprinting assays and live imaging at select loci, with or without small molecule perturbation of initiation using triptolide. Overall, studies that inhibit initiation, derive longer mean estimates of turnover rates (approx. 5–10 min) than studies that do not perturb initiation or use hypertonic shock to prevent Pol II recruitment (1–2 min). These differences could be attributed to the mechanism of actions and dynamics of triptolide function [55]. Still, the overall estimates of Pol II stability at the paused position are consistently longer than the initiation position.

**Figure 2.**
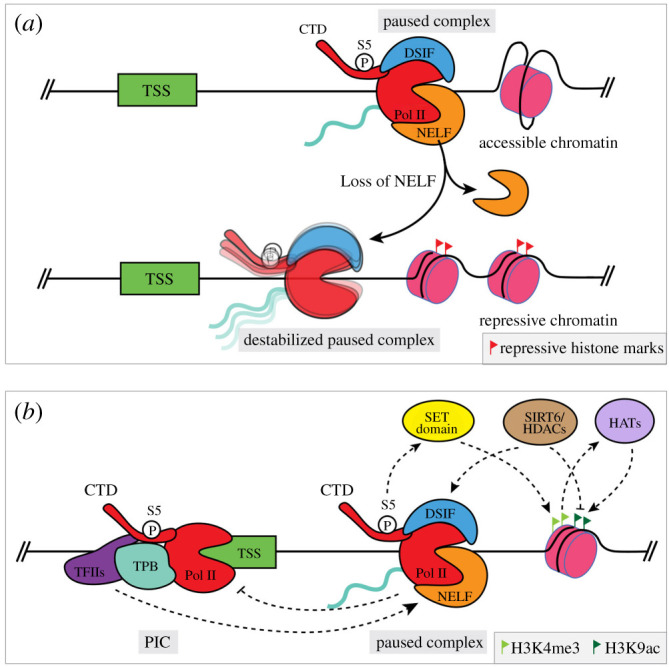
Paused Pol II interacts with its environment. (*a*) Loss of NELF results in a destabilized paused complex and consequently can lead to the establishment of a repressive chromatin landscape downstream. (*b*) Pol II pausing can interact with the PIC upstream and the chromatin landscape downstream through various histone modifiers to maintain or repress gene expression.

It remains unclear how Pol II pausing can directly influence overall transcriptional output from a particular locus. Several loss-of-function experiments of pausing components, such as the NELF complex, revealed that this can result in either up- or downregulated expression of highly paused genes in a variety of model systems [15,56,57]. The direction of change is likely to be dependent on the surrounding chromatin structure, since the presence of a paused complex has been suggested to maintain an accessible chromatin architecture at some promoters, ultimately resulting in higher overall transcription than would be possible without paused Pol II and an inaccessible chromatin [12,56] (figure 2*a*). However, since complete depletion of Pol II pausing components can take days to achieve (as in these studies), it is challenging to delineate gene loci directly affected by Pol II pausing from ones affected by secondary or compounding effects of these perturbations and the time scale of analyses. Still, these conclusions provide new and enticing avenues for investigating the endpoint consequences of perturbing pausing on the transcriptional activity of genes, and the overall transcriptional state of cells, even though they are limited by the tools employed, and thus lack direct experimental evidence.

### 2.4. Recent molecular insights into Pol II pausing

When considering a hypothetical gene, the Pol II pausing machinery lies near the PIC just upstream, with regulatory chromatin structure on the gene body downstream. Thus, it is fair to assume that these complexes might interact with each other. Upstream, with respect to the PIC, recent work suggests that the general transcription factor IID (TFIID) plays an active role in maintaining Pol II pausing via a direct or indirect interaction with the paused polymerase complex given their physical proximity (figure 2*b*). This interaction is abrogated when additional bases are added between the PIC site and pausing site [58,59]. This study used a novel, factor-defined transcription system to test this model *in vitro*, and a degron-based approach to test the hypothesis in cells. However, validation in cells is complicated by changes in transcriptional dynamics upon TFIID degradation that may be intrinsic to PIC assembly and pause-release rather than pausing. Therefore, the extent to which Pol II pausing might regulate new PIC assembly remains unclear, and conversely, feedback from the PIC itself onto the pausing machinery is yet to be evaluated. Nevertheless, these data strongly argue that while Pol II pausing may inhibit new Pol II initiation, it does not completely inhibit binding of other general PIC transcription factors which can keep a promoter ‘marked’ for activation.

It is also plausible that the pausing complex mediates chromatin accessibility and shapes the histone modification landscape around the TSS. The histone H3 lysine 4 trimethylation (H3K4me3) and lysine 9 acetylation (H3K9ac) modifications mark active promoters [60]. H3K4me3 is established by the methyltransferase activity of SET domain proteins, which are recruited by serine 5 phosphorylation of the CTD on paused Pol II [61]. The stability of the paused Pol II can in turn reinforce H3K4 trimethylation [62]. Subsequently, H3K4me3 marks facilitate H3K9 acetylation by recruiting histone acetyltransferases [60]. Thus, paused Pol II can facilitate the addition of H3K4me3 and H3K9ac modifications in the promoter region, which in turn enhance elongation via recruitment of the SEC [60]. On the other hand, histone deacetylases, such as SIRT6, can remove the H3K9ac mark, stabilize the paused Pol II complex and ultimately decrease the expression levels of target genes by controlling the assembly of P-TEFb and SEC [63] (figure 2*b*). A recent study suggests that other unidentified histone deacetylases may have a similar impact to SIRT6 on Pol II pausing [64]. These studies highlight the complex regulatory interactions of the pausing complex with other chromatin-associated factors, and bolster the theory that Pol II pausing can serve as a major determinant of overall gene transcription in a context-dependent manner.

## 3. RNA Pol II pausing in development

Faithful development of organisms requires coordinated and precise regulation of gene expression. Rapid responses to stimuli—both, extrinsic and intrinsic—dictate specific spatio-temporal gene expression patterns [65]. The resulting transcriptional states drive the diversification of cell types, and produce highly specialized and organized populations as the basis of organs and organ systems [66,67]. The essential roles of signalling cascades and transcription factors during development have been extensively characterized. Given the frequency of Pol II pausing and its effects on transcription, it probably also contributes to gene regulation in various developmental contexts. Indeed, Pol II pausing is required for development in *Drosophila*, zebrafish and mouse [16–18]. In this section, we discuss the current evidence of a role for Pol II pausing in development, particularly in mammals.

### 3.1. Pol II pausing in *Drosophila melanogaster* development

*Drosophila* has been the most extensively studied model for characterizing Pol II pausing [40]. NELF is maternally provided and its deletion results in failure of transcriptional activation in embryos and lethality shortly before the MBT and gastrulation [16]. Subsequent studies identified widespread Pol II pausing on most promoters during MBT, which coincides with the onset of zygotic transcription and establishment of the body plan [68,69]. ChIP-sequencing data revealed that Pol II is recruited to promoters of developmental genes ‘poised’ for activation. Additionally, these developmentally paused promoters share certain sequence features, such as the motif 10 element and pause button, suggesting an evolutionary use of promoter designs for developmental genes in a manner similar to other *cis*-regulatory elements that identify specific sequences. On the other hand, adult tissue-specific genes display Pol II pausing to a lesser extent, and tend to be driven by TATA box-containing promoters. The precise purpose of having these developmental genes paused prior to productive elongation has only been speculated. Since Pol II pausing is enriched at developmental and rapid response genes, it might aid in speed of activation, synchronized and co-regulated expression and the insulation of promoters from chromatin compaction [13,29,70].

### 3.2. Pol II pausing in mammalian development

In general, our understanding of Pol II pausing in mammalian systems lags that in *Drosophila*. This is due to the more complex nature of mammalian development and limited accessibility to large amounts of embryonic material. Nevertheless, loss-of-function studies in mouse embryos have revealed an essential role for NELF during early development. *Nelf* mutant embryos fail to progress beyond mid-gestation (approx. embryonic day (E) 8.0–9.0) [15,18], perhaps due to a failure of pluripotent epiblast cells to successfully differentiate in response to extracellular signals [18]. To characterize the defect in greater detail, these studies turned to the mES cell system.

mES cells can recapitulate many aspects of embryonic development, and thus have been used as a tractable and scalable model to study key developmental processes *in vitro* [71]. Pol II pausing has been shown to be prevalent in mES cells, with more than 40% of expressed genes showing a pausing index greater than 4 (that is, there are 4 times as many polymerases in the TSS ± 150 bases compared to the following 2 kb of the gene body) [15]. Of note, this quantitative limit of pausing index, four, is somewhat arbitrary, and reflects the lack of consensus for defining a specific threshold to use for deriving a pausing index. However, these studies still highlight the accumulation of paused Pol II complexes at a significant proportion of transcribed genes. While Pol II pausing in *Drosophila* is enriched at developmental genes, mES cells do not show a specific enrichment of Pol II pausing at developmental genes [15]. Human ES cells also show a similar proportion of paused genes, but it is not clear whether these gene classes are comparable to those in mES cells [72].

Manipulating Pol II pausing in mES cells via genetic deletion of *Nelfb*, a subunit of the NELF complex, revealed that while developmental and stem cell identity genes are unaffected, the genes which are mis-regulated coordinate responses to extracellular signals. By contrast to wild-type mES cells, *Nelfb* mutant cells are resistant to spontaneous differentiation. On a molecular level, transcriptional targets of FGF and WNT signalling pathways are affected, resulting in impaired downstream responses to these signalling cues [15,18,25]. These two pathways play crucial roles in balancing self-renewal versus differentiation in mES cells [73–75]. Therefore, these findings provide valuable insight into a functional role for Pol II pausing during mammalian development. Still, the molecular details of this role remain unclear—how does Pol II pausing regulate gene transcription in the context of cellular signalling, and the maintenance/disruption of self-renewal? Does the differentiation phenotype observed in mutant cells affect certain cell types and not others *in vitro*? Can the defects observed in mES cells shed light on the cause of lethality in embryos? As mentioned previously, interpretation of the results from studies in mES cells need to be de-coupled from potentially confounding secondary effects, such as cell cycle defects, which have been reported in these studies.

Other studies have also examined Pol II pausing in the context of regeneration via tissue specific NELF knockout in mice [76,77]. Mechanisms surrounding tissue regeneration often parallel developmental programmes, and therefore serve as surrogate models to study the Pol II pausing in regulating development. These studies specifically examined the endometrium and skeletal muscle. Both tissues share the requirement to frequently regenerate in the adult [76,77]. In both cases, loss of NELF did not have a significant impact on tissue homeostasis under steady-state conditions, but severely affected transformation/regeneration following injury. In the skeletal muscle, for example, NELF is required specifically for expansion of the muscle stem cell pool by enabling key responses to p53 and pigment-epithelium derived factor (PEDF) signalling [77]. These studies are in line with a proposed function for Pol II pausing in mediating response to signalling, and highlight a requirement for Pol II pausing during periods of cell identity specification and fate transitions.

### 3.3. Signalling and Pol II pausing: mediating a transcriptional response to stimuli

From the initial identification of the evolutionarily conserved Pol II pausing at heat-shock responsive genes from *Drosophila* to humans, genome-wide studies have highlighted that immediate-early release genes that respond to signalling pathways tend to be enriched for paused Pol II complexes [70,78–80]. This observation holds true for a variety of mammalian cell types, including human breast cancer cells (MCF-7), mouse primary macrophages and ES cells [15,18,79–84]. Recently, this was also observed in zebrafish, where Pol II pausing was shown to regulate hematopoiesis by controlling transcriptional targets of transforming growth factor beta (TGFb) signalling [17]. This conservation of enrichment at signalling-associated genes highlights a potential role for Pol II pausing in mediating and/or titrating the response to signalling pathways that ultimately guide cell fate decisions during development.

**Figure 3.**
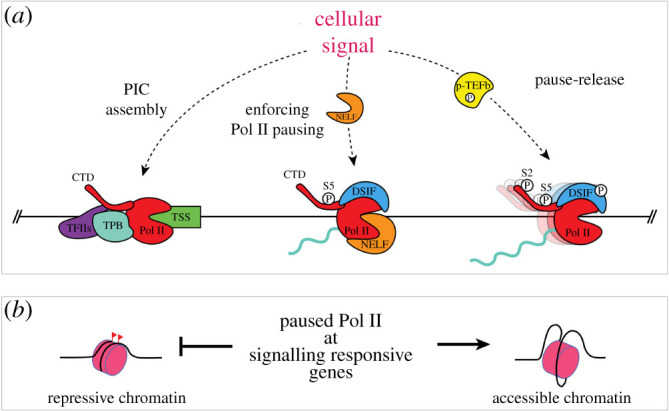
Points of interaction points between cellular signalling pathways and transcription. (*a*) Signalling cascades can regulate transcription at the level of PIC assembly, enforcement of Pol II pausing or pause-release. (*b*) Pol II pausing at inactive promoters downstream of signalling pathways can protect chromatin accessibility.

The role of Pol II pausing in mediating specific responses to cellular signalling was demonstrated prior to its proposed function in the context of development. In addition to the previously discussed signalling pathways, retinoic acid and oestrogen signalling can also modulate transcription specifically via control of Pol II pausing in mammalian cells, while disruption of this control is implicated in disease states such as cancer [81–85]. Oestrogen receptor alpha (ER*α*) directly interacts with NELFB and p-TEFB, and might mediate their recruitment to Pol II depending on signalling state (i.e. active or inactive) [81,82]. In mice, widespread NELF recruitment and release controls the inflammatory response in macrophages [83,84]. In several human cancer cell lines, stimulation of oestrogen signalling promotes ERα-mediated recruitment of NELF subunits specifically to ERα-associated promoters to attenuate signalling. By contrast, in MCF-7 cells ER*α* recruits P-TEFb to the *MYB* gene to drive transcription beyond the regulatory SL-dT region—a Pol II pausing site approximately 1.7 kb downstream of the TSS [82]. This dual function of pausing and pause-release emphasizes the crosstalk between signalling and Pol II pausing (figure 3*a*).

An alternative mechanism by which signalling may regulate Pol II pausing and pause-release is by controlling the activity of specific transcription factors. c-MYC, a pioneering transcription factor implicated in development and disease and controlled directly via the LIF/JAK/STAT signalling pathway, can regulate downstream gene expression primarily through pause-release and promoting active elongation in mES cells [27,86]. c-MYC directly recruits P-TEFb and other elongation factors to mediate pause-release [87]. Other transcription factors may associate with intermediate proteins to recruit P-TEFb. A similar example of such a mechanism has been noted in zebrafish, where the erythroid-specific TRIM33 transcription cofactor enables pause-release [36]. Of note, other transcriptional cofactors can also stabilize the paused Pol II and decrease overall expression, which has been shown to be the case for TRIM28 in human and mES cells [44]. It is yet to be determined if other transcription factors operating downstream of FGF, WNT and TGFb signalling might regulate Pol II pausing in a similar manner.

### 3.4. A case for Pol II pausing in early mammalian development

Until recently, the genomic landscape of Pol II binding during early mammalian development *in vivo* could not be examined due to limited biological material. By leveraging a transposase-coupled antibody assay with low input (approx. 500 cells), Liu *et al*. were able to map Pol II occupancy in pre-implantation mouse embryos [20]. They profiled the Pol II landscape from oocytes until the early blastocyst stage. The one-cell (zygote) and two-cell stages showed extensive Pol II pausing at active and inactive promoters, prior to zygotic genome activation (ZGA). This observation is reminiscent of Pol II pausing observed in early *Drosophila* embryos during the pre-MBT stage, which also coincides with ZGA [19,69]. Notably, Pol II pausing at inactive developmental genes becomes dramatically reduced between the two-cell stage (E1.5) and the blastocyst (E3.5), but is re-established soon after as observed in mES cells [20].

Altogether, these studies suggest that Pol II pausing may be required at distinct times in early mammalian development including two key stages—ZGA and EPI maturation and differentiation. The functional role of Pol II pausing during mammalian ZGA has not yet been assessed. By contrast, the requirement for *Nelfb* in early post-implantation development and mES cell differentiation are consistent with a role in the re-establishment of pervasive Pol II pausing at active and inactive promoters after the emergence of pluripotency in the blastocyst (i.e. specification of the EPI lineage ∼E3.5) [15,18].

Once pluripotency is established in the embryo, it is quickly disassembled as development proceeds. The pluripotency ‘continuum’ transitions from a ‘naive’ state in the blastocyst toward a ‘formative’ state in the newly implanted embryo before acquiring a ‘primed’ state as cells prepare for germ layer differentiation during gastrulation [88,89]. During this dynamic process, the cells of the pluripotent EPI lineage undergo a re-wiring of the transcriptional and enhancer circuitry to reflect this departure from the naive state [90–93]. Cellular signalling plays a major role in coordinating this transition as FGF, BMP, WNT and Nodal signalling pathways orchestrate pluripotency progression and gastrulation in the embryo [94,95]. FGF/ERK signalling is required in the mouse blastocyst for EPI maturation and exit from naive pluripotency [96–99]. This is generally achieved by attenuation of naive factor expression, such as *Nanog* and *Klf2* in the embryo as well as in mES cells, and genomic priming of formative markers, such as *Pou3f1*, *Lef1* and *Fgf5*, for efficient progression away from the naive state [90,100–103]. Similarly, careful regulation of the WNT and Nodal signalling pathways is also required to prepare the EPI for lineage priming and specification. While activation of WNT signalling promotes naive pluripotency *in vitro* by alleviating TCF3 (*Tcf7l1*) repression of naive pluripotent markers, its inhibition is required to maintain the primed state of pluripotency [75,104–109]. Activation of WNT signalling in this established primed pluripotent state induces further differentiation toward the mesoderm and endoderm lineages in the presence of Nodal signalling activity [110,111]. Timely regulation of Nodal signalling as pluripotency progresses is also required for efficient differentiation of mES cells into neural or ectodermal lineage versus mesendodermal lineages [89].

Considering that the establishment of pluripotency in the mouse embryo through to the initiation of gastrulation encompasses just three days of development, the reorganization of transcriptional networks and careful coordination of multiple signalling inputs in space and time must require a fine-tuned system of gene activation and repression. Since Pol II pausing has been reported in other mammalian and invertebrate systems as mediating rapid and coordinated transcriptional responses to signalling inputs, it may also function in a similar capacity during pluripotency progression and gastrulation. Loss of Pol II pausing in *Nelfb* mutant embryos results in embryonic lethality around the time of gastrulation and mutant mES cells display an attenuated response to differentiation cues, thus supporting such a proposed role for Pol II pausing during development [15,18,20]. Moreover, recent evidence that FGF/ERK signalling can mediate promoter and enhancer priming of loci associated with pluripotency transition in the naive state, and regulate Pol II binding at genomic loci further supports a probable function for pausing at these embryonic stages [92,103].

## 4. Potential roles for Pol II pausing in mammalian development

### 4.1. Rate of induction and/or repression of active elongation

There is evidence that Pol II pausing facilitates rapid induction of gene transcription, leading to the hypothesis that such genes might be primarily regulated at the level of pause-release with an inducing factor—a transcription factor or signalling effector—responsible for recruiting factors to release paused Pol II [28,29]. This mechanism of gene regulation seems plausible given that many transcriptional responses occur within minutes of signal induction [112,113]. Some of these genes, such as the heat shock responsive genes and some FGF/ERK targets, have already been shown to retain paused Pol II complexes in the absence of the inducing signal, and appear to be inefficiently expressed when pausing is destabilized [15,114]. However, the fact that NELF depletion in human DLD-1 cells does not affect the rate of induction of heat shock genes argues against this mode of action [115,116]. Furthermore, recent data show that Pol II recruitment occurs *de novo* at many rapidly induced FGF/ERK targets in mES cells upon signal induction [103]. These findings are yet to be expanded to other systems and inducing signals for further validation. However, the regulation of rapid gene expression is likely to be dependent on the cohorts of genes being considered, the cell state in question, the specific induction signal, as well as the types of promoter motifs present and epigenetic landscape surrounding the gene loci being activated.

**Figure 4.**
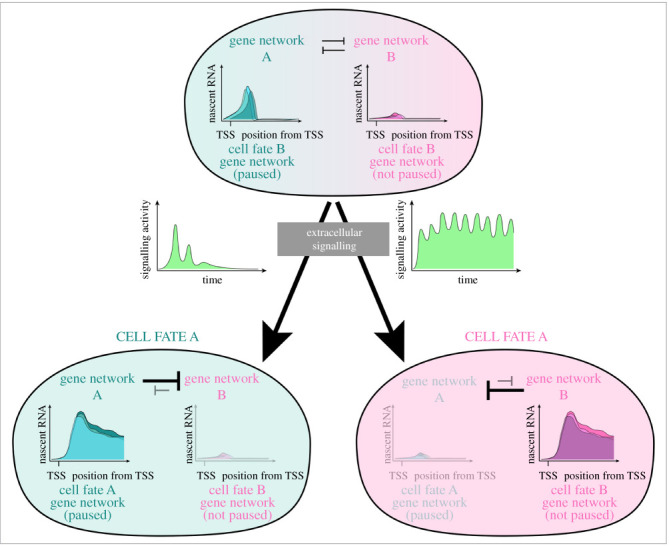
Proposed model showing how dynamic signalling activity can feed into paused Pol II to encode distinct cell fates. In models where a progenitor is specified to either cell fate A or cell fate B by different levels and/or dynamics of signalling activity, pausing can ‘prime’ the cell fate A gene network. A short temporal pulse of signalling can thus lead to pause-release and activation of gene network A and cell fate A specification. Mutual inhibition of opposing cell fate gene networks allows gene network A to repress cell fate B. Sustained signalling activity is required for sufficient induction of gene network B and establishment of cell fate B.

ERK activity in the mouse blastocyst is also known to be dynamic with subtle but specific differences between cell lineages driving the specification and later priming of the pluripotent EPI and mES cells [117,118]. Therefore, the question remains as to how such small differences elicit divergent responses in transcription and ultimately cell fate, and how transcription might keep up with the dynamic input of extracellular signalling. One might speculate that Pol II pausing can ‘poise’ genes downstream of ERK and other signalling pathways during pluripotency transitions. This priming could be established by known unphosphorylated ERK or other undefined intermediate factors. Such priming would allow at least a subset of ERK targets to be induced immediately coincident with a wave of ERK phosphorylation by favouring pause-release. Additionally, and perhaps more importantly, absence of pERK would result in re-enforcement of the paused Pol II, resulting in an immediate inhibition of active elongation. The well-studied heat shock response has been shown to display similar dynamics [115]. This level of control would enable cells to respond rapidly to dynamic signalling activity in both directions (on and off) and allow for fine-tuning of transcript dosage and transcriptional activity (figure 4). Eventually, the cumulative ERK dynamics, particularly the strength and duration of signal, will drive phased and specific transcriptional states that drive certain stages of pluripotency transitions. Molecularly, it is possible that pERK may drive immediate targets via repression of pausing factors such as NELF, or modulating the turnover rate of paused polymerases via release or termination.

### 4.2. Synchronous and tightly controlled induction of genes

In mammalian cells, synchronous induction of genes may be of particular importance for developmental signalling pathways that have several negative feedback loops, such as FGF/ERK and Nodal/Smad2 signalling, which can be induced rapidly to maintain specific levels of pathway activity and induce tissue morphogenesis [93,119,120]. In such pathways, negative feedback loops are established following the initial wave of signalling and induction of immediate targets. Disruption of the transcriptional dynamics of negative feedback-associated gene expression with respect to other target genes can amplify or attenuate signalling responses [121,122].

**Figure 5.**
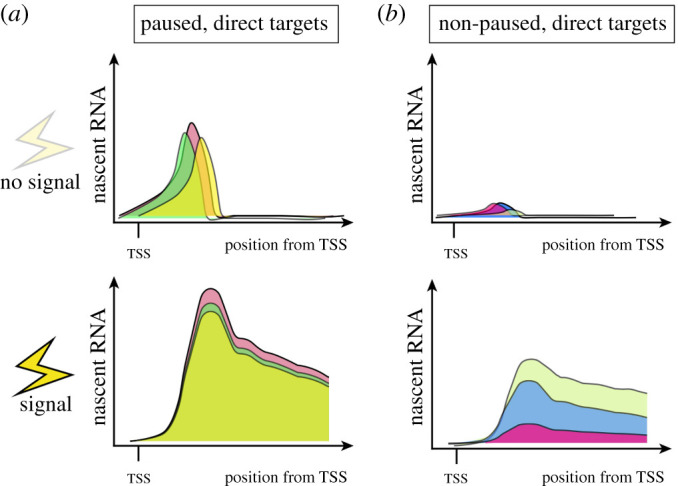
A model for synchronous induction of gene expression downstream of a signal mediated by Pol II pausing. Paused Pol II complexes at direct signalling target genes synchronously progress to productive elongation upon the inducing signal. Target genes lacking paused Pol II may have varying dynamics of transcriptional activation due to differences in chromatin accessibility and requirement of PIC assembly resulting in asynchronous induction. Different colours of nascent RNA tracks represent different target genes.

Synchronous gene induction not only requires paused transcription, but also strict regulation of Pol II pausing to prevent aberrant expression. Thus, Pol II pausing serves as a second ‘checkpoint’, along with Pol II recruitment, in a two-step control system to ensure robust induction without low or basal transcriptional activity (figure 5). The first step gives a signalling effector or transcription factor control over Pol II recruitment and PIC formation, while a second additional factor controls pause-release [13,70]. One example to support such a model comes from evidence, as previously discussed, that c-MYC primarily functions in controlling pause-release to activate genes in mES cells, while other pluripotency-associated factors have been shown to initiate transcription [27]. In mouse development, while FGF/ERK is required for specification of EPI and PrE lineages, other pathways, such as NODAL and NOTCH are active and have been shown to reinforce pluripotency *in vitro* and facilitate EPI maturation *in vivo* [89,123,124]. Certainly, it remains to be determined at which of the sequential steps of transcription these signalling pathways exercise control. It is plausible that these pathways could work with FGF/ERK to control recruitment of RNA Pol II and pause-release separately, particularly as the EPI matures and progresses from a naive pluripotent state toward the formative and primed states in preparation for gastrulation [74,125].

### 4.3. Marking active genes and enabling enhancer plasticity

Pol II pausing may also play a passive role in development simply as a function of its physical presence and kinetic stability relative to other components of the transcriptional machinery. In the mouse blastocyst, as EPI and PrE cells are specified and subsequently mature, their overall chromatin structures transition to a more compact, inactive state [126,127]. It would be necessary during these changes to keep promoters of active genes accessible for transcription. As discussed previously, depleting NELF to disrupt pausing can result in repressed transcription due to increased nucleosome occupancy in the vicinity of promoters [56]. Thus, paused Pol II possibly serves as a physical marker to keep active promoters vacant and protected from nucleosome occupancy (figure 3*b*).

**Figure 6.**
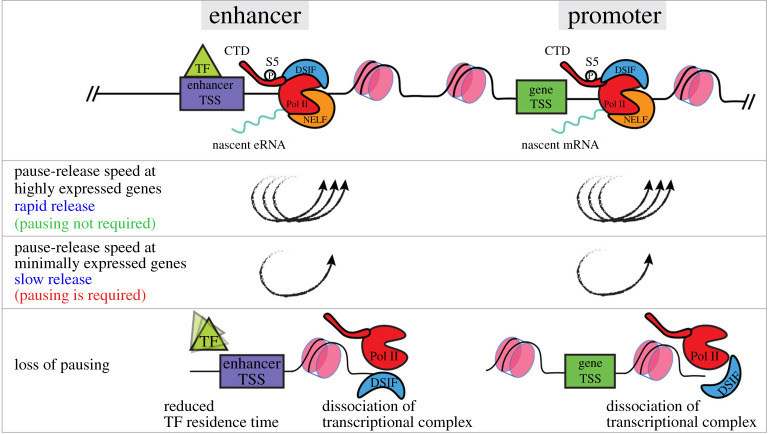
Proposed model outlining the contribution of Pol II pausing at promoters and enhancers to overall regulation of gene expression. Pol II pausing occurs at gene promoters as well as enhancers in mES cells. At highly expressed genes, paused Pol II is short lived at both promoters and enhancers and has a rapid turnover. In this case, Pol II pausing can be a rate-limiting step for gene expression. However, at minimally expressed genes Pol II pausing may serve necessary roles in preserving expression where it is has a longer half-life. In the absence of Pol II pausing, TF residence time at enhancers can be drastically reduced leading to destabilization and dissociation of the transcriptional complex. Similarly, the transcriptional complex is destabilized at the gene promoter leading to downregulation of expression.

Recently, widespread Pol II pausing was also detected at actively transcribed enhancers in *Drosophila* S2 and mES cells [47]. It is yet to be determined whether the protein complexes responsible for Pol II pausing at enhancers are the same as those near gene promoters, and whether Pol II pausing at enhancers and associated gene targets are coordinated. However, given that the residence time of transcription factors on enhancer DNA is estimated to be on a time scale of milliseconds to seconds [128–130], having polymerases paused for longer time scales (order of minutes) could drastically increase the stability of enhancer function by ensuring enhancer transcriptional activity irrespective of the rapid binding-dissociation dynamics of transcription factors. Furthermore, Pol II pausing may have a key role at minimally expressed enhancers or genes by serving as a molecular ‘tab’ between sparse TF binding events to extend the plasticity of these loci and ultimately direct cell fate. These proposed roles for Pol II pausing are in accordance with recent data showing that naive pluripotency factors can remain bound to enhancers hours after the cells are induced to exit pluripotency, and after the associated target genes are downregulated [103] (figure 6).

While Pol II pausing may keep promoters and enhancers protected from nucleosomes and accumulation of repressive histone marks, the molecular mechanisms for how this is accomplished have yet to be characterized. It is possible that the pausing complex prevents promoters from being closed between ‘transcriptional bursts' when Pol II complexes are not actively transcribing. These bursts are a well-documented phenomenon of mammalian transcription, occurring minutes to hours apart—a time scale that allows for some chromatin modifications and rearrangements [4,131,132]. This means that while transcriptional bursts involve rapid pause-release, maintaining some paused Pol II would be essential to keep a promoter open and active between bursts, particularly at promoters with low bursting frequency [133,134]. This model is supported by the finding that developmental enhancer loops are mostly stable across tissues and developmental stages in *Drosophila*, and remain associated with paused polymerases at target gene promoters [135]. Alternatively, the pausing complex may recruit additional factors to maintain chromatin accessibility and transcriptional activity by promoting the formation or maintenance of transcriptional condensates near promoters [1,136,137].

Addressing these possible roles for Pol II pausing in the context of mammalian development would improve our understanding of transcriptional regulation and successful establishment of resulting cellular states. Moreover, it may help explain how cells translate extrinsic or intrinsic stimuli for rapid and coordinated transcriptional responses driving key differentiation and morphogenetic processes that constitute developmental progression.

## 5. Technical limitations and new avenues

It is important to discuss the experimental approaches that have been used to make the conclusions discussed so far. Studying global transcription dynamics has been facilitated by advances in nascent RNA sequencing techniques allowing detailed identification of sites of Pol II pausing and kinetics [12,13]. By contrast, directly manipulating Pol II pausing has been achieved primarily using classical genetic approaches, such as knockout of the NELF complex subunits. Small molecule inhibitors can also be used to target transcription initiation and elongation [12,13,29]. Although these inhibitors have been valuable in studying the dynamics and kinetics of pausing, drawing conclusions about the specific function of pausing from these manipulations can be problematic due to non-specific effects on factors aside from those within the pausing complex [37,138]. Meanwhile, genetic approaches to specifically target Pol II pausing factors can also be limiting because of their inherent time scale of action. For example, effective knockout or knockdown using CRISPR-Cas9 or RNAi requires a time scale of hours to days to achieve between inducing the change and assaying the loss-of-function phenotype [15,18,56,57]. While this is not necessarily an impediment, for contexts where cell survival or proliferation are affected, it can result in confounding secondary effects that mask the immediate function of paused Pol II in regulating gene expression. Indeed, upon NELF depletion via conditional knockout or RNAi, several mammalian cell lines including mES cells, mouse embryonic fibroblasts and the human DLD-1 cancer cells cease to proliferate within a few days, making it challenging to interpret the functional phenotypes reported in these studies [15,18,139,140].

More importantly, transcription is a process that proceeds on a time scale of seconds to minutes [50,51]. To test the function of Pol II pausing and couple molecular mechanisms with specific phenotypes, one must be able to assess the impact of destabilizing Pol II pausing acutely, within a similar time scale of transcription itself. It is challenging to rapidly modulate protein levels with tools that manipulate expression at the level of DNA or RNA. Fortunately, these limitations can be circumvented using novel tools for rapid protein degradation [141–144]. These approaches involve tagging endogenous proteins with short peptides which, in the presence of a small molecule, induce ubiquitination and proteasomal degradation. These techniques can achieve acute depletion of target proteins, usually within minutes to hours, and have already been used to refine the molecular functions of NELF and Spt5 in human DLD-1 cells [116,145,146]. Combining such tools with the high resolution of nascent RNA-seq techniques to study the functional relevance of pausing in contexts such as response to developmental signals could reveal hitherto uncharacterized roles of Pol II pausing on transcription and cellular states.

RNA Pol II pausing has emerged as a widespread phenomenon in metazoans, and a potential node for gene regulation in mammalian systems. There is a growing list of molecular interactions and functions of the pausing complex, yet it has been challenging to attribute cellular phenotypes to molecular perturbations of Pol II pausing. In particular, Pol II pausing is critical to organismal development from *Drosophila* to mice, with increasing evidence that it provides a crucial link between cellular signalling inputs and transcriptional outputs during a variety of cellular state transition events. With recent high-resolution analyses of Pol II pausing in early mammalian development and tools for rapid perturbations, the door is open to dissect the specific functions of Pol II pausing in mammalian development.

## Data accessibility

This article has no additional data.
